# Smoking and non-allergic sinonasal disease

**DOI:** 10.1186/2045-7022-3-S2-O11

**Published:** 2013-07-16

**Authors:** Vibeke Backer, Christian von Buchwald, Kåre Håkansson, Simon Francis Thomsen, Allan Linneberg

**Affiliations:** 1 Bispebjerg Hospital, Dept of Respiratory Medicine L, Copenhagen, Denmark; 2 Copenhagen University Hospital, Dept of Head and Neck Surgery, Copenhagen, Denmark; 3 Copenhagen University Hospital, Copenhagen, Denmark; 4 Glostrup Hospital, Centre for Prevention and Health, Copenhagen, Denmark

## Background

The association between smoking and lower airway inflammation and disease is well documented; however, it is not established whether smoking also induces disease of the upper airways. In a previous study, we found non-allergic rhinitis (NAR) to be associated with smoking in a dose-dependent manner; in addition, asthma and chronic bronchitis were linked with NAR. In the present study, we re-test the hypothesis that smoking can cause non-allergic sinonasal disease.

## Method

A cross-sectional study of a random population sample (n=3762; age, 18--69 years) was conducted in Copenhagen, Denmark. Study subjects were invited to a general health examination that included questions about airway diseases, a skin prick test (SPT) to common aeroallergens, and measurement of pulmonary function; 1522 (40.5%) persons accepted. For further analysis, we divided the population into the following groups: (I) negative SPT and persistent symptoms of sinonasal disease lasting more than four weeks (non-allergic chronic rhinosinusitis); (II) positive SPT and rhinitis (allergic rhinitis); (III) no rhinitis with or without positive SPT (background).

## Results

We found that non-allergic chronic rhinosinusitis in comparison with the background group was associated with ever smoking (odds ratio [OR] = 1.60 [1.00--2.55]), asthma (OR = 2.52 [1.35--4.90]) and chronic bronchitis (OR = 2.26 [1.26--4.04]). Mean spirometric values were not significantly decreased in any group. The association with chronic bronchitis was stronger in non-allergic chronic rhinosinusitis than in allergic rhinitis, whereas the opposite was observed for asthma.

## Conclusion

This study confirms that both smoking and chronic bronchitis are associated with non-allergic sinonasal disease. We conclude that smoking, at least in some cases, can be a triggering factor for the development of non-allergic sinonasal disease.

